# Paving the future: finding suitable ISMB venues

**DOI:** 10.1093/bioinformatics/bts420

**Published:** 2012-07-13

**Authors:** Burkhard Rost, Terry Gaasterland, Thomas Lengauer, Michal Linial, Scott Markel, B.J. Morrison McKay, Reinhard Schneider, Paul Horton, Janet Kelso

**Affiliations:** ^1^International Society for Computational Biology, 9500 Gilman Drive, MC 0505 UCSD/SDSC, La Jolla, CA 92093-0505, USA, ^2^TUM, Department of Informatics, Bioinformatics & Computational Biology - i12 & Institute of Advanced Study (TUM-IAS) & WZW Weihenstephan, Boltzmannstr. 3, 85748 Garching/Munich, Germany, ^3^New York Consortium on Membrane Protein Structure (NYCOMPS) & Department of Biochemistry and Molecular Biophysics, Columbia University, 701 West, 168th Street, New York, NY 10032, ^4^Scripps Institution of Oceanography and Institute for Genomic Medicine, University of California, San Diego, 9500 Gilman Drive, MS0202, La Jolla, CA 92093 USA, ^5^Max Planck Institute for Informatics, Saarbruecken, Germany, ^6^Hebrew University, Jerusalem, Israel, ^7^Accelrys Inc., San Diego, USA, ^8^Luxembourg Centre for Systems Biomedicine, University of Luxembourg, Luxembourg, ^9^AIST, Computational Biology Research Center, Tokyo, Japan and ^10^Max Planck Institute for Evolutionary Anthropology, Leipzig, Germany

## Abstract

The International Society for Computational Biology, ISCB, organizes the largest event in the field of computational biology and bioinformatics, namely the annual international conference on Intelligent Systems for Molecular Biology, the ISMB. This year at ISMB 2012 in Long Beach, ISCB celebrated the 20th anniversary of its flagship meeting. ISCB is a young, lean and efficient society that aspires to make a significant impact with only limited resources. Many constraints make the choice of venues for ISMB a tough challenge. Here, we describe those challenges and invite the contribution of ideas for solutions.

**Contact:** assistant@rostlab.org

***ISMB evolved*.** The largest scholarly meeting event in computational biology is the annual conference of the International Society for Computational Biology, the ISCB, namely the international conference on Intelligent Systems for Molecular Biology (ISMB). The ISMB meeting series began in 1993 in Bethesda (Maryland, USA) and aimed to connect computational experts to address challenges in molecular biology. That first meeting was so successful that it spawned a series of annual conferences with increasing participation. The 8th ISMB (in 2000 at UCSD in San Diego) was the first ISMB that brought together over 1000 participants. Due to its increased size, it was also the last ISMB that could be held at a university. The 12th ISMB [and the first joint meeting with the European Conference on Computational Biology (ECCB) in Glasgow, 2004] attracted over 2000 attendees. At that point, the concept of a single track with only about 50 talks was no longer sustainable, and ISMB went parallel, beginning with a double track. The 14th ISMB in Fortaleza, Brazil was the last time that most of the organization was shouldered significantly by local scientists.

Beginning with the 15th ISMB (and the 2nd ISMB/ECCB) in Vienna in 2007, ISCB took full responsibility for the organization. It has been a great challenge for ISCB to move from the position of what was essentially an ‘advisory observer’ to that of completely managing the event over the course of six meetings (2007–2012). Taking the leading role in the organization of the conference provides ISCB with many possibilities to simplify and to evolve a ‘signature style’. As a result, the organization of each new ISMB has increasingly moved toward an orchestration of tasks carried out autonomously, each of which follows some specific template and is driven by track chairs. Conference organization is guided by a Steering Committee (SC) composed of the meeting and track chairs, staff and key contractors. The committee holds biweekly telephone conferences during which organizational issues are discussed and decisions taken.

ISMB involves the participation of over 2000 scientists; it runs on a total budget exceeding 1.5 million dollars; and it was presented for the 20th time this year in Long Beach, CA. Those who contemplate these three numbers may assume that the ‘new’ organizational structure is an evident necessity. This might seem so, but the realization of this structure was by no means straightforward.

***Today: massively parallel, big event*.** Today ISMB is an event of impressive proportions. This leads to immense challenges in every respect. Other conferences/societies meet many of those challenges, but no other society with limited resources similar to those of ISCB successfully takes on such a particular combination of tasks. Meetings of comparable complexity and size typically operate on a budget that is significantly more comfortable. In other words, they spend much more money and involve several times the staff.

At the ISMB in Long Beach (July 15–17, 2012), we realized a total of approximately 1600 participants. As is now customary for ISMB, a number of smaller special interest groups (SIGs) and satellite meetings were organized prior to the main ISMB conference. Some of these are, in their own right, the most important annual meetings for particular research areas. The 6 keynote addresses were followed (morning) or preceded (afternoon) by a 2-h session of 4 talks within each of 9 parallel events/tracks, for a collective total of over 200 talks presented during the 3-day main meeting. Care is taken to maximize the slots for presentations without reducing the open spaces that allow participants to meet. Guiding criteria are: starting at 9 a.m. and ending at 5:30 p.m.; reserving a 2-h break for lunch; and providing open blocks of time for meeting with colleagues. In particular, maintaining the open blocks of time is particularly difficult given that ISMB is the major event in the field, not only due to the number of participants, talks and the impact on the field, but also through the participation from the most visible representatives of the field: a significant fraction of the top groups are present at each ISMB. This means that the venue is densely packed with an immense amount of creative potential, which also means that many want to piggyback additional special events upon the main regular schedule. Put differently, it is difficult to ‘protect’ any free time—but also essential. Indeed, a schedule that makes space for meeting informally with colleagues has become a signature of ISMB.

***How well do room sizes fit?*** The structure of ISMB implies several important constraints for the choice of a venue: we currently require that the venue have one room that comfortably seats over 1500 participants, and there should be at least four other rooms that can seat a considerable fraction for the multi-track sessions. The specification of ‘considerable fraction’ remains tricky, and the larger and more numerous the rooms, the more expensive the venue rental. The challenge is to strike just the right balance between rental costs and optimal space, and to do so when the final attendance is still very much an unknown.

ISMB/ECCB 2004 in Glasgow remains by many measures the most successful meeting in the history of ISMB and ECCB, and the positive collaboration that now exists between these two groups started with that one conference. In addition to being our first joint event, it was also our first time presenting two parallel papers tracks (as a combination of long and short papers), plus tracks for demos and SIG meeting reports. Needless to say, we were experimenting with expansion options, but we underestimated the attendance that our first joint meeting would draw, and had not anticipated just how popular any given talk within a multi-track conference could be. Due to the requirements imposed by the number of parallel sessions, there is no practical way to change rooms at the last minute if a particular talk or session is in such high demand that it exceeds the capacity of its reserved space. We learned the hard way that if the size of a session room is too small for even one talk in that session, too many attendees will become frustrated. Glasgow paved the way for working together successfully and thinking boldly outside the box. It also continues to serve as a reminder that we must be careful when reviewing space limitations in otherwise ideal venues.

Choosing venues with rooms for many parallel sessions that can each accommodate over 350 delegates not only increases the venue rental cost, but it also means that ISMB is restricted to the very few conference centers that have such facilities. These conference centers are typically able to accommodate considerably larger meetings of over 20 000 delegates–a very different league! We needed to find a way in between the Scylla of ‘big single track conferences’ and the Charybdis of ‘car exhibitions’.

The ISMB venues visited since we have gone massively parallel were all ideal in many respects. In order to find these ideal venues in Vienna, Toronto, Boston, Stockholm and Long Beach, ISCB had to discard many others. Typically, ISCB receives quotes from six to eight venues/cities, and visits two or three of these sites based on financial viability. Quotes collected by the ISCB conferences director, Steven Leard, for meeting venues in London, New York and Paris, for example, were significantly beyond the financial reach of the Society. Simply finding a suitable venue for ISMB is increasing in effort, and it was with this in mind that the decision was taken to experiment with returning intermittently to ideal conference venues. Repetition of sites has the additional advantage that, as a repeat customer, it should allow the negotiation of better prices for rental and services, and that it allows ISCB to develop a relationship with the local service providers. The first repetition, Vienna (2007 and 2011) was only partially successful in that the price had increased between the two events beyond expectation. This might exclude Vienna from future ‘returns’. The second return will be Boston (2010 and 2014) that will hopefully become the first ‘safe’ site to which ISCB can return at least once every 4 to 8 years.

The main requirements are: one room with over 1500 seats that can be filled by the participants without too much acoustic disruption (a seemingly simple constraint that is not satisfied by all venues visited), a number of additional rooms seating between 250 and 500 participants, and another handful with 50–150 seats. Seating should be able to be emptied and filled in less than 5 min during the brief break between all talks that is synchronized for all tracks. Further, the 500–1000 posters should be placed such that the probability of being visited is high; and exhibitors should have enough space that is in a central location passed regularly by each delegate. The degree to which we achieve this is directly proportional to the success of attracting exhibitors who are crucial for the success of the meeting.

These current specifications still restrict the number of venues that can host an ISMB. All venues visited since 2007 could have hosted ISMBs with more than three times as many participants. The additional costs for hosting more participants at a venue are typically small. Put differently, the larger ISMB, the lower the per-participant cost which could translate to lower registration fees and a more secure future for ISCB.

***Optimizing attendance*.** Eighty percent of ISCB's members work in North America and Western Europe. Not surprisingly, analysis of participation statistics show that European members are much more likely to attend meetings in Europe, and North Americans are much more likely to attend those in North America. The venue should therefore be as easy as possible to reach for as many participants as possible. This is supported by the observation that the number of North Americans attending meetings in Europe, and Europeans attending meetings in North America, has increased since ISCB has been held in venues that are most easily reached from both sides of the Atlantic. Obviously, this excludes many world regions with large and active computational biology communities. An attempt to address this issue was the initiation of a series of ISCB-x meetings (ISCB-Africa, ISCB-Asia, ISCB-Latin America) that provide an ISCB meeting in regions not typically visited by ISMB.

ISCB and ECCB agreed to co-host their meetings in the years when ISMB is in Europe. We attempt to maximize attendance by choosing venues as close as possible to major airline hubs that serve as international entry ports. Identification of a suitable place for ISMB also involves additional dimensions such as ease of obtaining visas for travel. As a point in case, for citizens from many countries, it is easier to enter Canada than to enter the USA. Nevertheless, holding ISMB in Canada means that members who work in the USA with passports from countries that require visa renewal upon re-entry are unable to attend without the risk of a delayed or denied return after the conference.

***Soft criteria: optimize success*.** Beyond the criteria mentioned above, there are many other important issues to consider when choosing ISMB venues. Essentially, all of those revolve around the degree to which a venue furthers the contentment of attendees and the extent to which it satisfies those involved in organizing the meeting.

An important issue is the accessibility of the venue from the airports/train stations as well as from the core of most frequented hotels [in particular from the headquarter hotel (HQ hotel)]. Conversely, for the choice of the HQ hotel, it is important how close this is to general sites of interest for delegates. Attractive locations also leave a positive memory and increase the odds of returning to other ISMBs (over 70% of all participants do return).

The environment of the venue and/or the city must be able to easily accommodate approximately 2000 participants. Many conferences are organized in a hotel and all participants stay in that hotel. This allows for the organizers to negotiate better conditions in exchange for reserving a large block of rooms. Not so for ISMB: almost half of the ISMB participants need accommodations that are more affordable than can be negotiated with hotels that feature conference facilities. Reserving a large block of rooms comes with a substantial risk of ISCB having to pay for any rooms that have not been used. Therefore, the block that ISCB reserves with local hotels is relatively small in relation to the amount of conference space required for the meeting. This conservatively low number of contracted rooms results in some cities/hotels showing less interest in our conference due to the low rooms-to-space ratio.

Given the venue size that ISMB now needs, planning 1 year ahead, as was done in the past, is insufficient. Indeed, for the Vienna 2007 meeting, site visits were conducted almost 2 years in advance. It was, however, only at the point when the ISCB Board approved the return to successful venues that contracts were prepared well in advance: ISCB now has signed contracts for the Berlin 2013 and Boston 2014 meetings, and we have already been working on identifying sites for the meetings in 2015 and 2016.

The search for suitable venues for 2015–2016 has been complicated by economic challenges. Venues are becoming more expensive while funding for science continues to stagnate. Given this, it is difficult to counter the increased costs by raising registration fees (fee were last increased in 2007).

The attractiveness of the venue–city combination contributes importantly to the perception of each ISMB's success. The most important soft criteria for any ISMB is that the meeting should optimally support the scientific success, should formidably trigger the dialogue and exchange between scientists, and should contribute favorably to the growth and interests of ISCB members. Put most simply: the happier the participants, the stronger the conference becomes for the community as a whole. ISMB venues must be positively memorable, must be efficient and practical, and their surroundings must be pleasant.

A major challenge for all venues we have visited to date has been the availability of sufficient internet bandwidth. Despite the fact that a convincing team handling IT is one selection criterion, and that ISCB provides detailed requirements for IT infrastructure to the selected conference centers, every venue since 2007 has realized within the first hours of the main meeting that the internet demands of ISMB are substantially higher than they typically encounter, even as compared with what they considered to be their most high-demand high-tech past conference clients. Computational biology is a trendsetter in Internet, data and computer needs. Even in 2011, very few large venues were fully up to this challenge.

***How to proceed?*** All venues chosen since 2007 navigated on favorable winds through the optimization problem sketched above. The five venues in Vienna, Toronto, Stockholm, Boston and Long Beach stood out against twice as many others that were not chosen. Each of those five had its own tradeoffs with respect to the needs of ISMB.

Each of those venues also could have hosted many more participants. Instead of being full, the meetings of 2007–2011 had ample additional spaces we could have filled. But more importantly, the proceeds from that span of years provided ISCB with just enough revenue to run at or slightly below our break-even point. To dream in the most extreme way: had 5000 scholars attended each ISMB since 2007, the Society could now double its staff, triple its activities to benefit members and still be financially secure for 50 years. Alternatively, the registration fee for all participants could have been halved without losing revenue.

Most scientists generally prefer small workshops to large conferences because small meetings increase the odds of meeting new colleagues in relaxed environments. The main ISMB meeting (excluding satellite meetings) has now been condensed to 3 days; it is preceded by 2 days of SIG meetings, the Student Council symposium and tutorials. At ISMB/ECCB 2011 in Vienna, we had 14 such pre-conference meetings. It has been proposed that the SIG meetings could be scaled up to have 10×14 such meetings spread over a longer period. However, ISCB simply does not have the human resources to do this. In fact, ISCB now handles one immensely complex ISMB and six other meetings with almost the same staff it had before it got intensively involved with the organization of any meeting other than ISMB.

Nevertheless, ISCB recognizes the needs of its members that would benefit from more specialized meetings. Diversification has been one answer to the challenges: ISCB now is managing a total of seven different meeting series that lead to six meetings each year: ISMB (bi-annual), ISMB/ECCB (bi-annual), ISCB-Latin America (bi-annual: 2010, 2012, 2014), ISCB-Africa-ASBCB (bi-annual, 2011, 2013), ISCB-Asia (annual: 2011, 2012), Rocky (annual, since 2003), CSHALS (annual, since 2008) and GLBIO (annual, since 2011). Other meetings are currently in the concept or evaluation stage and will likely follow as early as next year.

The new meetings and other activities contribute importantly to the growth and impact of the Society, to its scientific success, and ultimately to the growth in membership. Financially, the six non-ISMB meetings organized by ISCB collectively provide 25% of the total proceeds of ISMB. As it stands, ISCB's members continue to benefit from a successful, thriving ISMB.

We, the ISCB Executive Committee and ISCB Conferences Committee Chairs, are writing this contribution to raise awareness and with the motivation of increasing the base of those who understand that your presence at ISMB is one important way in which you can support your profession through participation in the primary activities of your professional Society. ISCB members thrive at ISMB and benefit from its longevity and continuation. ISCB also needs new meetings, new activities, new adventures that satisfy our community's thirst for information, for education and for connecting with one another. For all of that, ISCB needs your presence, your support and your passion! We welcome your ideas and suggestions for how to proceed.

